# Mechanical and Adhesive Properties of Hydrothermally Treated Bamboo Composites Reinforced with Phenolic Resin: Effect of Impregnation with Silica Nanoparticles

**DOI:** 10.3390/polym17222989

**Published:** 2025-11-11

**Authors:** Lionnel Frederique Bidzanga Bessala, Yanjun Li

**Affiliations:** Bamboo Industry Institute, Zhejiang A&F University, Hangzhou 311300, China

**Keywords:** bamboo composite, phenolic resin, silica nanoparticles, hydrothermal treatment, viscoelasticity, adherence

## Abstract

This study investigates the synergistic effect of phenolic resin impregnation on the mechanical and adhesive properties of hydrothermally treated bamboo composites further reinforced with a silica nanoparticle sol–gel catalyzed by Fe_3_O_4_ (SiO_2_/Fe_3_O_4_). The hydrothermal pre-treatment was found to enhance cellulose crystallinity, as confirmed through XRD analysis. Dynamic mechanical analysis (DMA) and nanoindentation tests revealed that the hybrid treatment significantly influences the viscoelastic response. Composites treated only with hot water and resin (GB-W) exhibited superior short-term creep resistance and higher elasticity, attributed to their optimized crystalline structure. In contrast, the silica-reinforced composites (GB-M) demonstrated the most viscous behavior and lowest stress relaxation, making them most effective at minimizing elastic springback. Nanoindentation further showed that GB-W had the highest nano-adherence at the fiber cell wall level. FTIR analysis indicated a stronger interaction between the phenolic resin and the hydroxyl groups of the bamboo matrix in GB-0 and GB-W compared to GB-M, where the silica layer potentially altered this interface. Microscopy confirmed a resin penetration depth of at least 1 mm, primarily into porous tissues. The results demonstrate that while silica reinforcement enhances relaxation properties, the hydrothermal pre-treatment combined with phenolic resin creates a more favorable interface, leading to better overall creep resistance and adherence.

## 1. Introduction

Bamboo, a renewable natural material, exhibits promising industrial potential due to its rapid growth and exceptional mechanical properties [1,2,3,4,5]. Like most lignocellulosic materials, its ductility can be improved through hydrothermal treatments [6,7,8,9,10,11,12]. Moderate hydrothermal treatment induces a complex multi-scale response in bamboo. Although it facilitates bending, this treatment leads to an irreversible reduction in the modulus of elasticity, even after drying. To circumvent this trade-off and improve the material’s performance for structural applications, various reinforcement methods have been explored.

A well-established strategy to counter this softening is impregnation with phenolic resins [13]. The choice of phenolic resins as a binding and reinforcing agent for bamboo is supported by several demonstrated advantages. Firstly, resin-impregnated bamboo pieces can form durably cohesive assemblies under certain conditions. For example, converting the surface of natural bamboo to an oxidized bamboo surface can significantly increase the bonding strength of bamboo. This strength can be further improved if dowels are added [14,15,16,17,18]. Furthermore, studies indicate that the water absorption of treated bamboo decreases proportionally with pre-impregnation drying temperatures, reported in the literature to be between 50 °C and 70 °C, while its hardening is directly correlated to this temperature [19,20,21]. Impregnation with a phenolic resin significantly enhances the mechanical properties of bamboo: it increases bending and impact strength, improves toughness, and optimizes shear strength [20,22]. Moreover, during hot pressing, the resin promotes the densification of bamboo cells, reducing the risk of cracking—a phenomenon proportional to the resin content [23]. Additionally, curved bamboo pieces, whether resin-reinforced or not, are often assembled using various fasteners, the most common being metal dowels and pins [17,18,19]. The holding power of these pins in bamboo can be characterized by its adherence and, at the microscopic level, by nano-adherence.

An alternative approach using mineral impregnation with an iron (III) oxide (Fe_3_O_4_)-catalyzed silica (SiO_2_) sol–gel offers a different set of advantages [24,25,26,27,28,29]. This approach not only allows for the restoration of the modulus of elasticity, potentially even beyond the initial value [27], but could also improve the fungicidal durability of bamboo [28,29]. More significantly, by interacting with bamboo’s fundamental structural polymers, it offers the potential to optimize the balance between stiffness and viscoelastic damping. Given this complementary potential of two distinct approaches—the proven binding and reinforcing capability of phenolic resins versus the promising viscoelastic modulation and mineralization offered by Fe_3_O_4_-catalyzed SiO_2_—a synergistic strategy is therefore of great interest. However, to date, few studies have examined the influence of phenolic resins on the properties of hydrothermally pretreated bamboo reinforced with a magnetic silica sol–gel, particularly at the fiber level—key elements of its mechanical performance.

Consequently, the long-term goal is to reconcile the benefits of phenolic resin reinforcement with those of SiO_2_/Fe_3_O_4_ nanoparticle additives in reinforcing bamboo pieces bent using hydrothermal methods. However, within the scope of this study, the focus will be on characterizing the effect of a hybrid SiO_2_/Fe_3_O_4_ and phenolic resin system on the viscoelastic and adhesive properties of hydrothermally softened bamboo. Specifically, this work will evaluate the impact of the phenolic resin on the mechanical (adherence, short-term creep and relaxation, modulus of elasticity) and chemical (penetration depth, bond formation with hydroxyl groups) properties of bamboo that was first hydrothermally softened and then modified with Fe_3_O_4_-catalyzed silica nanoparticles.

## 2. Materials and Methods

### 2.1. Materials

The following chemicals were supplied by Macklin (Shanghai, China): tetraethyl orthosilicate (TEOS, 98% purity, 208.33 g/mol), hexadecyltrimethylammonium bromide (CTAB, 99% purity, 364.45 g/mol), absolute ethanol (46.07 g/mol), purified iron(III) oxide (97% purity, 231.53 g/mol), and an ammonium hydroxide solution (25–28% concentration, 35.05 g/mol). The alcohol-based thermosetting phenolic resin BR2130 was obtained from Henan Jinrun New Materials Co., Ltd., Gongyi, China; this resin had a solid content greater than 70%, water content less than 2%, and a free phenol content of approximately 10%.

### 2.2. Sample Preparation, Thermal Treatment and Resin Coating

Moso bamboo (Phyllostachys edulis) strips, aged 3–4 years and harvested from culms at a height of at least 1.2 m, were used to prepare the specimens. Initially, standardized slats measuring 300 mm × 20 mm × 8 mm were cut and planed. Various samples were then obtained from this base portion. All samples were oven-dried at 103 °C for 48 h until anhydrous and then immediately packaged in aluminum foil to maintain a constant moisture content. They were stored for 24 h prior to the thermal and chemical modifications.

For nanoindentation, 5 mm × 5 mm × 8 mm cubes were produced, each featuring a 4 mm-high pyramidal cone machined into the 8 mm thickness. Following this preparation, two main batches were established.

- Batch GB-0-N underwent a treatment protocol beginning with brush-coating of the conical surface using thermosetting phenolic resin BR2130. Immediately following coating application, samples were transferred to a vacuum oven where simultaneous resin impregnation and curing occurred under a residual pressure of 10 mbar at 130 °C for 4 h, ensuring complete integration of the resin into the bamboo matrix.
- An additional hot water treatment was applied to some samples: immersion in pure water at 70 °C for 15 min. This specific temperature and treatment duration were selected based on observations of bamboo bending techniques employed by several Chinese companies. This treatment led to the creation of batch GB-W-N, which was treated with the resin according to the same protocol as GB-0-N after immersion. For each sample, 10 points were evaluated by nanoindentation.

For DMA (dynamic mechanical analysis) tests, specimens measuring 40 mm (length) × 12 mm (width) × 2 mm (thickness) were prepared by thinning, sectioning, and bonding. Specifically, one series of samples was obtained by bonding two 40 mm × 12 mm × 1 mm elements together using the thermosetting resin. The bonding procedure involved coating the 40 mm × 12 mm faces of the samples with a brush. The two coated faces were then brought into contact and inserted between the metal plates of a press (UNLONG, model LH-JA, Xiamen, China). The assembly was subjected to a constant pressure of 10 MPa, applied to both faces, at a temperature of 130 °C for 30 min, then transferred to a vacuum oven where simultaneous resin impregnation and curing occurred under a residual pressure of 10 mbar at 130 °C for 3 h and 30 min. Each batch of samples contained 3 specimens and the DMA samples prepared this way were divided into:

- GB-0: Resin impregnation and hot pressing at 130 °C for 30 min, followed by vacuum drying for 3 h and 30 min.
- GB-W: Hydrothermal treatment (immersion at 70 °C for 15 min), followed by resin impregnation, pressing, and drying according to the same protocol as GB-0.

In parallel, for chemical modification, a dispersion was prepared by adding 100 mg of purified iron(III) oxide powder (magnetite, Fe_3_O_4_, 97%) to a mixture of 100 mL absolute ethanol and 10 mL pure water. Then, 100 mg of hexadecyltrimethylammonium bromide (CTAB) was introduced as a surfactant, and the mixture was homogenized by ultrasonication for 30 min. Following this, 1 mL of ammonium hydroxide solution (25–28%) was gradually introduced under magnetic stirring at 400 rpm to catalyze the hydrolysis and condensation reactions of TEOS by creating a basic medium, which led to the formation and gelation of a silica network within the bamboo microstructure. The stirring speed was then increased to 600 rpm, and 5 mL of tetraethyl orthosilicate (TEOS, 98%) was added dropwise over 5 min. Finally, several drops of ammonium hydroxide were added to adjust the pH to approximately 9. Bamboo samples, previously hydrothermally treated in pure water at 70 °C for 15 min to a moisture content of about 24%, were immediately immersed in this solution. The system was maintained at 70 °C for 15 h to allow for the complete infiltration and formation of the silica layer and its fixation within the bamboo matrix. After the reaction, the modified bamboo samples (GB-M-N, for nanoindentation and GB-M for DMA) were immediately coated and inpregnated with adhesive under the same conditions as GB-0 and GB-W for DMA samples and as GB-0-N and GB-W-N for nanoindentation samples.

### 2.3. Energy Dispersive X-Ray Spectroscopy (EDX)

To verify the effectiveness of silica network infiltration and iron within the bamboo matrix, an analysis using a scanning electron microscope attached with energy dispersive X-ray spectroscopy (SEM-EDX) was performed. This analysis utilized a Hitachi SU8010 (Tokyo, Japan) scanning electron microscope. The samples were taken from the GB-0, GB-W, and GB-M specimens, which were previously prepared for dynamic mechanical analysis (Section 2.2) with dimensions of 40 mm (length) × 12 mm (width) × 2 mm (thickness). It should be noted that these specimens were fabricated by bonding two 40 mm × 12 mm × 1 mm elements together using a thermosetting resin. From these specimens, 6 mm × 6 mm × 2 mm samples were extracted from the center using an ultra-thin sectioning machine. These samples were then coated with a thin layer of gold prior to observation. The SEM-EDX analysis was conducted on a 1 mm thickness to characterize the silica penetration. The acquisition was performed under an accelerating voltage of 15 kV. The acquisition parameters included a dwell time of 0.5 ms per point, an amplification time of 3.84 μs, and a single frame recorded using an OCTANE PLUS detector (Ametek, Berwyn, PA, USA), all in Region of Interest (ROI) mode. The data analysis consisted of comparing the sum of the silicon (Si) and iron signal intensities over a 1 mm distance to determine which sample contained the greatest quantity of silica and iron.

### 2.4. DMA and Mathematical Models of Short-Term Creep and Relaxation

**Figure 1:**
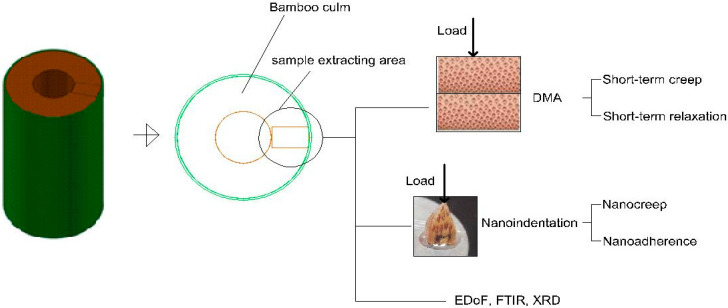
Bamboo sample extraction and test analysis.

Short-term creep and relaxation measurements were carried out using a Dynamic Mechanical Analyzer, DMA Q800, TA Instruments (New Castle, DE, USA), equipped with a dual-cantilever sample holder. Creep curves were recorded at a constant temperature of 45 °C. To minimize the influence of microcracks on the assessment of creep and relaxation on the dynamics under study, a constant load equivalent to 13% of the MOR (Modulus of Rupture) was applied according to the mode described in Figure 1, that is, perpendicularly from the zone of large-diameter fibres towards the zone of small-diameter fibres, with a span of 35 mm, and maintained for 20 min. Meanwhile, relaxation curves were obtained by maintaining a constant strain of 0.1% at 45 °C for 20 min.

The fractional Maxwell model makes it possible to describe, by means of a power law, the real behavior of bamboo in creep and relaxation. It has the advantage of requiring a reduced number of parameters to model the short-term viscoelastic response. Furthermore, the physical interpretation of these parameters remains relatively intuitive, justifying its choice for this study. The fractional Maxwell model establishes that if σ, the creep stress applied to the bamboo, is independent of time; E is the elastic modulus of the bamboo; η is the viscosity coefficient of the bamboo; t is the creep duration; and α is the order of the fractional derivative of strain with respect to time t; then the creep strain of the bamboo as a function of time is determined by Equation (1) [30].

(1) $$\varepsilon \left(t\right)=\frac{\sigma }{E}+\sum_{k=1}^{n}\frac{\sigma }{\eta^{\alpha_{k}}}\cdot \frac{{\left(t-t_{k-1}\right)}^{\alpha_{k}}}{\Gamma (1+\alpha_{k})}$$

where Γ is the complete gamma function; with $\alpha =\alpha \left(t\right)=\alpha_{k}$, k being a natural integer; thus, for k=1, $t=t_{1}$; for k=2, $t=t_{2}$ etc. The elastic modulus during the creep test is determined by dividing the creep stress by the initial strain of the bamboo, i.e., when t = 0.

If the strain is constant and the stress varies as a function of time, it is a case of relaxation, and Equation (1) can be rewritten as Equation (2).

(2) $$\frac{\sigma (t)}{\varepsilon }=G(t)=\frac{1}{\frac{1}{E}+\sum_{k=1}^{n}\frac{1}{\eta^{\alpha_{k}}}\cdot \frac{{\left(t-t_{k-1}\right)}^{\alpha_{k}}}{\Gamma (1+\alpha_{k})}}$$

where G(t), expressed in MPa and varying as a function of time, is the relaxation modulus.

The creep and relaxation parameters of the fractional Maxwell model are therefore the modulus of elasticity E, the viscosity coefficient η, and the order of the fractional derivative of stress or strain α. Note that the modulus of elasticity is none other than the relaxation modulus of the bamboo in its initial state, i.e., when t = 0.

### 2.5. Nanoindentation

The adherence, creep, and elasticity of fibers from different bamboo samples were determined by nanoindentation. For this purpose, nanoindentation measurements were performed using a three-sided Berkovich tip mounted on an iMicro microindenter (KLA Corporation, Milpitas, CA, USA) equipped with an InForce 1000 electromagnetic actuator. The system recorded indentation depth as a function of both time and applied load. Two tests were performed.

**Figure 2:**
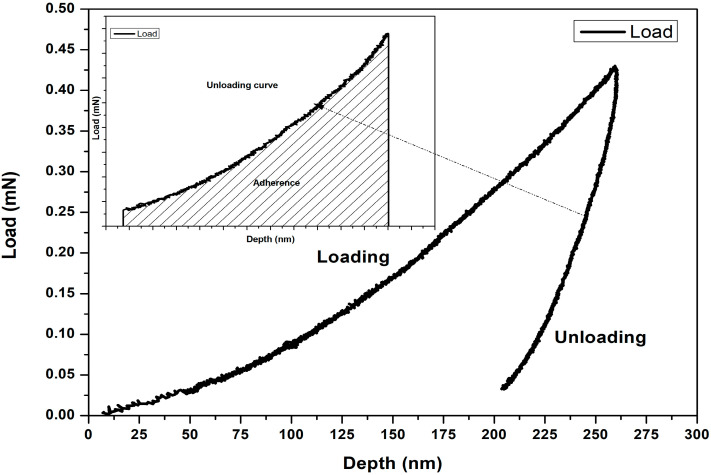
Measurement principle of adherence by nanoindentation.

In the first test, related to the measurement of nano-adherence (adherence work or debonding energy), the load was gradually increased to 0.40 mN and then immediately reduced until the tip was fully retracted (zero load). This protocol, consisting of a loading phase followed by an unloading phase, allowed for the analysis of fiber Adherence, defined as the energy required to reduce the load from 0.40 mN to 0 mN. Mathematically, this energy corresponds to the area under the depth-load curve during the unloading phase (Figure 2). The unloading curve was fitted with a third-degree polynomial using IBM SPSS Statistics 21 software, and its integral was calculated and evaluated between 0.40 mN and 0 mN for each bamboo type using Equation (3).

(3) $$A=\int_{0}^{0.40}L\left(h\right)dh$$

where A is the adherence in mN·mm, L is the load in mN, and h is the depth in mm. The cubic fit was chosen because, among the various fitting options provided by the software, it yielded the highest correlation coefficient, with a value greater than 0.99.

**Figure 3:**
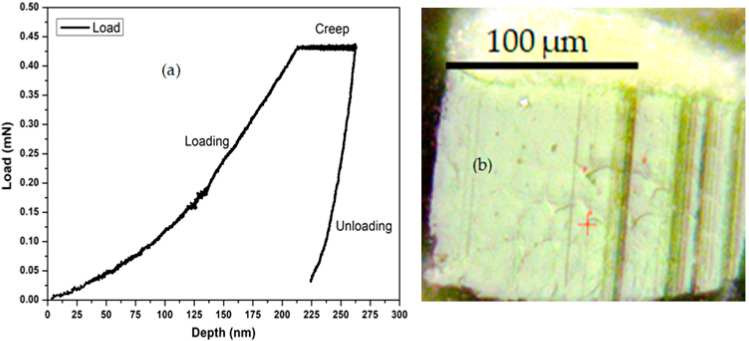
Short-term nano-creep model of bamboo. (**a**): test results; (**b**): Location of the indentations on the fiber’s microstructure. The red dots mark the indented areas. The red cross indicates the cursor position during image capture. indentation.

The second test consisted of measuring the creep of the bamboo fiber cell wall. The creep behavior was determined by analyzing the variation in indentation depth over time under a constant load (Figure 3a). The nanoindentation creep parameters were as follows: surface approach velocity of 100 nm/s, creep load of 0.40 mN, and creep duration of 200 s. This nanoindentation creep test consisted of three stages: loading, constant load holding, and unloading. For both tests, all measurements were performed on a number of points varying between 3 and 10 (Figure 3b).

In nanocreep analysis—where creep properties are measured via nanoindentation—the yield strength (P) of bamboo can be estimated using Tabor’s relation [31]. This empirical principle links yield strength to the material’s hardness (H), as determined by nanoindentation:

(4) $$P=\frac{H}{C}$$

(5) $$H=\frac{L}{A_{C}(D)}$$

where P is the indentation load, Ac is the contact area (which is a function of the indenter’s penetration depth), and C is Tabor’s constant. During the nanocreep phase, the load P is held constant and the contact area Ac varies only slightly. Under these steady-state creep conditions, both P and the hardness H are time-independent, leading to the relation P·C = H. Furthermore, the stress beneath the indenter ($\sigma_{nano}$) is approximately equal to the yield strength, resulting in the following Equation (6).

(6) $$\sigma_{nano}\cdot C\approx H$$

The connection between the elastic modulus and yield strength, as measured by both DMA and nanoindentation, is established using a fractional Maxwell model. The model’s elastic modulus (E), which corresponds to the bamboo’s instantaneous strain (at t = 0), is directly comparable to the nanoindentation-derived modulus (Equation (7)) [32].

(7) $$Enano=\frac{\sqrt{\pi }}{2\beta }\cdot \frac{S}{A}$$

In Equation (7), S (stiffness) is the slope (dP/dh) of the initial portion of the unloading curve in a load-displacement plot, A is the projected contact area, and β is a geometric correction factor.

The fractional Maxwell model is adapted for nanoindentation analysis by substituting the theoretical strain, ε(t), with the experimentally measured indenter penetration depth, D(t), in the bamboo. Correspondingly, the conventional elastic modulus, E, is replaced by the reduced elastic modulus, $E_{nano}$. To incorporate the material’s yield strength, an equivalent nanoindentation stress, $\sigma_{nano}$, is defined using Tabor’s relation (P = H/C). This calculated $\sigma_{nano}$ is then inserted into the model’s creep equation, resulting in Equation (8). This methodology enables the fractional Maxwell model to be precisely calibrated against nanocreep data, thereby extracting the nanoscale viscoelastic parameters (η, α).

(8) $$\;D\left(t\right)=\frac{\sigma_{nano}}{E_{nano}}+\sum_{k=1}^{n}\frac{\sigma }{\eta^{\alpha_{k}}}\cdot \frac{{\left(t-t_{k-1}\right)}^{\alpha_{k}}}{\Gamma (1+\alpha_{k})}$$

where the summation is from k = 1 to n.

### 2.6. Extended Depth of Field Microscope (EDoF)

From the bonded samples (GB-0, GB-W, and GB-M) and a control sample (B-C: raw, unbonded bamboo), prisms measuring 6 mm (length) × 6 mm (width) × 2 mm (thickness) were cut (from the bond line for GB-0, GB-W, and GB-M) and characterized by staining. Specifically, GB-0, GB-W, GB-M, and B-C were fully immersed in an aqueous methylene blue solution for 30 min, and then partially bleached for 45 min in a 6% sodium hypochlorite solution. After rinsing with water and a 24 h stabilization period, the stained surface of each sample was observed across a 1 mm thickness using a high-depth-of-field microscope (Keyence VHX-1000, Keyence, Osaka, Japan) in HDR mode with lighting conversion. This selective bleaching step enhanced the contrast between lignin, which bleaches more rapidly, and the resin (which bleaches less rapidly than lignin), thereby improving image sharpness for a qualitative evaluation of adhesive penetration.

### 2.7. XRD

Bamboo powder was obtained by grinding and then sieving to retain the particle size fraction between 80 and 100 mesh. After this sieving, the samples were dried to an anhydrous state. Using an X-ray diffractometer (BRUKER, D2 phaser, Bremen, Germany), the intensity of the X-ray radiation was measured as a function of the diffraction angle. Subsequently, the degree of crystallinity was estimated using the empirical Segal method (peak height method) [33], which involved comparing the intensity of the main crystalline peak to the minimum intensity between the crystalline peaks, which is assumed to represent the amorphous contribution. The degree of crystallinity (C_2_θ), expressed as a percentage, was evaluated for each sample using Equation (9). This calculation method involves relating the integrated intensity of the crystalline peaks (I_2_θ) to the total intensity, after subtracting the amorphous background estimated at the minimum (I_min_).

(9) $$C_{2\theta }(\%)=\frac{I_{2\theta }-I_{min}}{I_{2\theta }}\times 100$$

### 2.8. FTIR

The bamboo samples were ground into powder and sieved, then vacuum-dried at 80 °C for 12 h. Powders with particle sizes larger than 100 mesh were used for FTIR testing (Thermo Nicolet 6700, Thermo Scientific, Waltham, MA, USA). Infrared spectroscopy of the powder samples was performed in ATR mode to analyze changes in functional groups of specimens before and after chemical treatment. The scans were performed with 64 accumulations at 4 cm^−1^ resolution, and IR spectra were acquired in the wavenumber range of 4000 cm^−1^ to 400 cm^−1^.

## 3. Results and Discussion

### 3.1. Cristallinity

**Figure 4:**
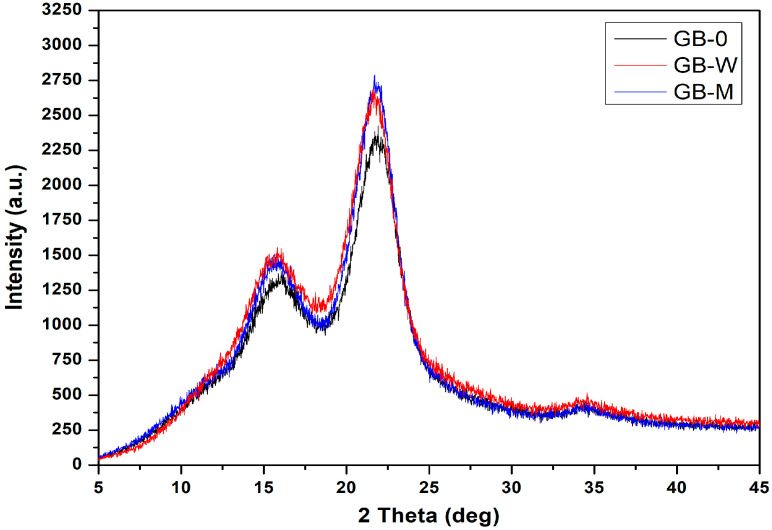
X-ray radiation intensity as a function of the 2θ angle for bamboo samples with resin impregnation.

The X-ray diffractograms (Figure 4) of the bamboo samples GB-0, GB-W, and GB-M display the characteristic profile of cellulose Iβ, with two main peaks located at 2θ ≈ 15° and 2θ ≈ 22°. The intensity minimum observed at 2θ ≈ 18.5° is attributed to the amorphous phase, primarily composed of hemicelluloses and lignin [34].

Analysis of Table 1 highlights the evolution in crystallinity of the bamboo samples GB-0, GB-W, and GB-M, based on the observed diffraction peaks. For the GB-W sample, the impact of the treatments is contrasting. A significant increase in crystallinity is observed at the peak at 2θ = 15°, where the value rises from 12.88% (GB-0) to 21.24%. This increase can be attributed to the hydrothermal treatment, which reorganizes the cellulose chains in the less ordered regions. In contrast, at the main peak at 2θ = 22°, which reflects the most crystalline domains, the crystallinity only increases marginally, from 53.56% to 54.79%. This result indicates that the hydrothermal treatment and resin impregnation modulate the reorganization process, limiting the increase in crystallinity in the already highly structured areas [35]. In striking contrast, the GB-M sample exhibits a much more effective synergy between the treatments. Its crystallinity at the main peak (2θ = 22°) reaches 63.84%, a value significantly higher than that of GB-0 (53.56%) and GB-W (54.79%). This remarkable performance is explained by a two-step interaction: on one hand, the hydrothermal treatment initiates a massive reorganization in the less ordered regions, as evidenced by the more than doubled value at the 2θ = 15° peak (27.91% for GB-M vs. 12.88% for GB-0). On the other hand, the impregnation with phenolic resin, potentiated by the presence of nanocomposites, rigidifies the entire matrix. This combined action stabilizes and amplifies the crystalline structure, leading to a significant improvement in both the disordered regions and the most organized crystalline domains.

### 3.2. Short-Term Creep and Relaxation

A comparative analysis of the resin-impregnated composites (GB-0, GB-W, GB-M) reveals that their mechanical behavior in creep and relaxation is dictated by the interaction between the pre-treatment and the microstructure of the bamboo matrix, as evidenced by XRD analyses.

**Figure 5:**
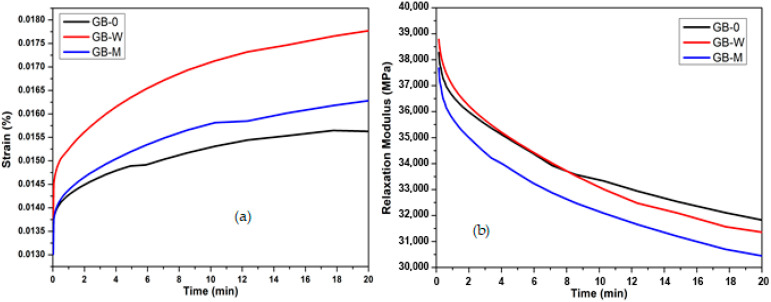
Short-term creep (**a**) and short-term relaxation (**b**) of resin-impregnated bamboo.

Creep Performance (Figure 5a, and Table 2): The GB-W composite, resulting from a hot-water pre-treatment, exhibits the most elastic creep behavior, as indicated by its highest α exponent (0.295 compared to 0.235 for GB-0). This property is attributed to the high cellulose crystallinity induced by the hot-water treatment, revealed by XRD analysis. However, this composite shows the lowest viscosity (η^α^) (3452 MPa·min^α^), indicating a reduced viscous resistance to creep. The GB-M composite, treated with oxides, possesses the highest modulus of elasticity (38,423 MPa). Its creep behavior, characterized by an α exponent of 0.265 and a viscosity of 3920 MPa·minᵅ, is better than that of GB-0 but less elastic than that of GB-W. XRD analysis suggests this is linked to its higher crystallinity compared to GB-W [36].

The Relaxation Behavior (Figure 5b, and Table 2), crucial for minimizing springback, shows a complete reversal of trends. The GB-M composite emerges as the best performer, displaying the lowest α exponent (0.538) and the lowest η^α^ viscosity (805,271 MPa·min^α^). This reflects the most viscous behavior and the lowest propensity for springback. The microstructure optimized by the oxide treatment, which promotes efficient energy dissipation, explains this performance. Conversely, GB-W exhibits a higher α (0.584) in relaxation, suggesting that the hot-water pre-treatment, although beneficial for crystallinity and creep, creates a bamboo–resin interface less conducive to relaxation than the chemical treatment.

### 3.3. Nanocreep and Nanoadherence

**Figure 6:**
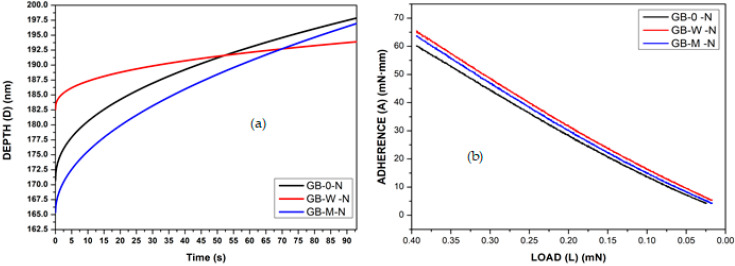
Cell wall fiber short nano-creep (**a**) and nanoadherence (**b**) of different bamboo samples.

The analysis of behavior at the microscopic (Figure 6 and Table 3) and macroscopic scales is clarified in light of the hydrothermal pre-treatment common to all modified samples, which induces an increase in cellulose crystallinity. This foundational structural effect allows for the isolation of the specific impact of subsequent additives. The emerging profiles reveal that the optimization of this pre-structured cellulose depends crucially on the nature of the treatment.

Indeed, the oxide treatment appears to reconfigure this initial potential differently. For GB-M, the addition of oxides to the pre-crystallized cellulose results in the most elastic cell wall (α = 0.506). However, this high microscopic elasticity is not fully translated to the macroscopic scale, where GB-M is outperformed by GB-W in terms of elasticity (α = 0.265 vs. 0.295) and creep resistance. This suggests that at the macroscopic level, the oxides in the impregnated composite might limit the optimal expression of the properties conferred by the crystalline cellulose, perhaps by affecting the fiber/resin interface [20]. Thus, the performance gap of GB-M compared to GB-W is not due to the absence of a hydrothermal treatment, but rather to the nature of the additive used thereafter. The hot-water pre-treatment creates a structural potential that the phenolic resin (GB-W) exploits more effectively than the metal oxides (GB-M) to develop a composite with superior viscoelastic properties, particularly in creep resistance (Table 3).

The comparative analysis of the adherence functions, based on Figure 6b and Table 4, demonstrates that the GB-W sample possesses the best adherence, requiring the highest withdrawal energy for the indenter [20]. This is followed by the GB-M sample, which exhibits intermediate adherence, while the GB-0 sample requires the lowest withdrawal energy [37,38], thus indicating the least effective adherence to the bamboo fiber cell wall.

### 3.4. Resin Penetration in Bamboo

**Figure 7:**
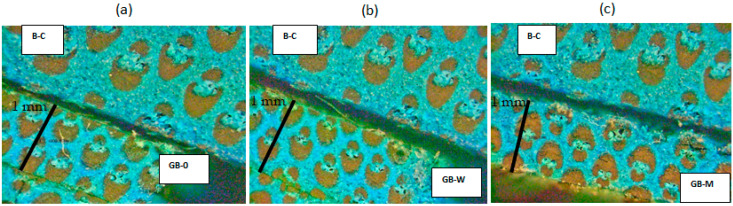
Color comparison of methylene blue-stained bamboo samples GB-0 (**a**), GB-W (**b**), GB-M (**c**), and the B-C bamboo sample.

The staining of lignin with methylene blue appears under extended depth-of-field microscopy (EDoF) set in ‘HDR + Lighting Conversion’ mode as a blue-green hue [39], while phenolic resin shows a light blue coloration [40]. Observations reveal that, like methylene blue, the phenolic resin primarily impregnates the parenchyma and vascular tissues of bamboo (Figure 7). In contrast, fibrous tissues exhibit weak or even absent staining, confirming that phenolic resin interacts minimally with the crystalline cellulose of the fibers. Moreover, the resin only marginally penetrates the bamboo fibers. Instead, it preferentially infiltrates highly porous structures, such as parenchyma and vascular bundles [41]. It was also observed that, for all samples (GB-0, GB-W, and GB-M), the resin impregnated a minimum depth of 1 mm. Indeed, the characteristic light blue staining of the resin was detected throughout the sample thickness (2 mm), corresponding to an effective penetration of 1 mm, since two impregnated 1 mm-thick samples were bonded edge-to-edge before analysis.

### 3.5. Affinity of Different Bamboo Types with Phenolic Resin

**Figure 8:**
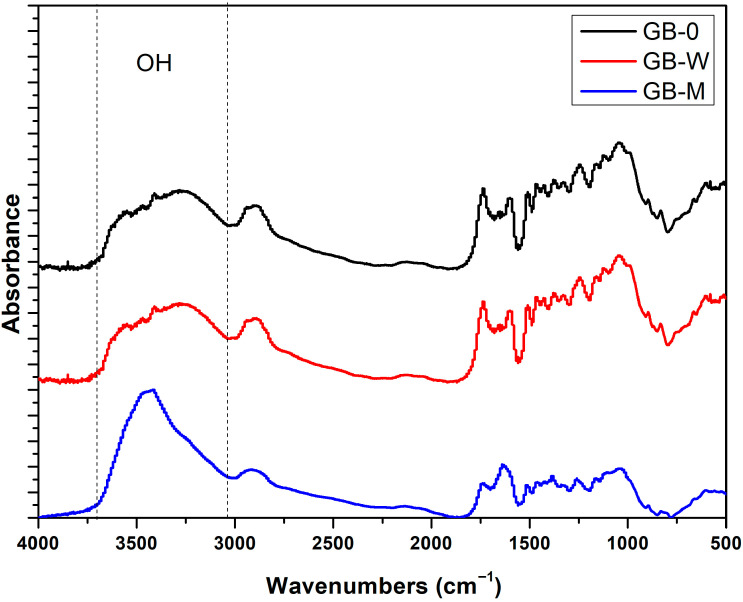
FTIR spectra of GB-0, GB-W and GB-M.

In Figure 8, the broad absorption band between 3000 cm^−1^ and 3700 cm^−1^ corresponds to the stretching vibrations of hydroxyl (O–H) groups. A critical observation is the shift of this band to a lower wavenumber, around 3200 cm^−1^, for the GB-0 and GB-W samples, whereas it remains centered at ~3400 cm^−1^ for the GB-M sample. This shift is a key indicator of the formation of strong hydrogen bonds between the phenolic resin and the hydroxyl groups of the bamboo’s structural polymers in GB-0 and GB-W. The higher wavenumber and greater intensity of the O-H band in GB-M suggest a predominance of ‘free’ or weakly bonded hydroxyl groups. This is consistent with the presence of a silicon dioxide (SiO_2_) layer in GB-M, which acts as a physical and chemical barrier that impedes the direct and optimal hydrogen bonding between the resin and the lignocellulosic matrix [36]. Consequently, the bamboo in samples GB-W and GB-0 exhibits superior interfacial affinity and chemical bonding with the phenolic resin compared to GB-M.

### 3.6. Infiltration of the Silicate Network and Magnetite into the Bamboo Matrix

**Figure 9:**
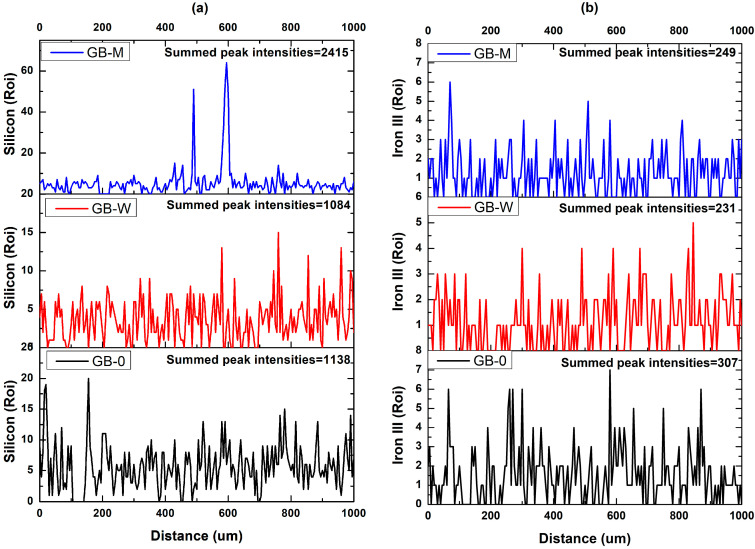
EDX spectrum of silicon (**a**) and magnetite (**b**) from the different bamboo samples.

The EDX analysis along a 1 mm profile (Figure 9) demonstrates the efficacy of the chemical treatment. The GB-M sample, treated with TEOS-magnetite, shows significant silica infiltration, with high-intensity peaks (exceeding 50 Roi) between 490 and 600 µm, resulting in a total silicon intensity sum of approximately 2415 Roi. This is 50% higher than both GB-0 (1138 Roi) and GB-W (1084 Roi). Regarding magnetite infiltration, the cumulative peak sum for the control GB-0 is 307, establishing a baseline level attributed to natural impurities in the bamboo [42]. The GB-W sample, subjected to hydrothermal treatment, shows the lowest sum at 231, indicating a leaching phenomenon of natural ferrous compounds by the hot water. In contrast, the value for the GB-M sample, treated with TEOS and magnetite, is 249, representing a slight superiority of 8% compared to GB-W. This increase suggests that the sol–gel reaction medium enabled some retention of the iron oxide particles, partially counteracting the leaching effect.

## 4. Conclusions

This study compared two reinforcement strategies for hydrothermally softened bamboo: impregnation with phenolic resin alone versus a hybrid system combining phenolic resin and SiO_2_/Fe_3_O_4_ nanoparticles. The findings clearly demonstrate that the hydrothermal pre-treatment effectively increased cellulose crystallinity. When followed solely by phenolic resin impregnation (GB-W composite), this process yielded a material with good short-term creep resistance, high elasticity, and superior energy dissipation at the fiber level. In contrast, while the incorporation of silica nanoparticles (GB-M composite) successfully enhanced viscous-dominated behavior—beneficial for reducing stress relaxation and elastic springback—it concurrently impaired the chemical affinity and interfacial bonding with the phenolic resin. This led to overall inferior creep performance compared to GB-W. The superior properties of GB-W are attributed to a more favorable interface and stronger bonding, likely through hydrogen bridges, between the resin and the bamboo’s hydroxyl groups. Consequently, for applications demanding high-dimensional stability under load and robust interfacial adhesion, the combination of hydrothermal pre-treatment and phenolic resin impregnation is a more effective strategy than the additional incorporation of silica nanoparticles under the studied conditions. Future work should focus on modifying the silica–matrix interface to overcome the observed chemical incompatibility and unlock the full potential of the ternary system.

## Figures and Tables

**Table 1:** Cristallinity for different bamboo samples.

2θ (°)	GB-0 XRD Intensity (a.u.)	Cristallinity (%)	GB-W XRD Intensity (a.u.)	Cristallinity (%)	GB-M XRD Intensity (a.u.)	Cristallinity (%)
15	1157	12.88	1408	21.24	1358	27.91
18.5	1008	-	1109		979	
22	2164	53.56	2453	54.79	2707	63.84

**Table 2:** Maxwell’s fractional creep and relaxation model parameters for different bamboo samples.

Tests	Samples	MOE (MPa)	α	$\eta^{\alpha }$ (MPa·min^α^)	Initial Strain (%)
Creep	GB-0	38,284	0.235 *	4464 *	0.01306
	GB-W	36,405	0.295 *	3452 *	0.01373
	GB-M	38,423	0.265 *	3920 *	0.01301
Relaxation	GB-0	38,274	0.591 *	981,892 *	-
	GB-W	38,780	0.584 *	807,662 *	-
	GB-M	38,423	0.538 *	805,271 *	-

* the linear regressions used to determine these parameters had correlation coefficients greater than 0.95.

**Table 3:** Maxwell’s fractional creep parameters for different cell wall bamboo fibers.

Samples	MOE (MPa)	α	$\eta^{\alpha }$ (MPa·min^α^)
GB-0	12,664	0.453 *	0.864 *
GB-W	8992	0.389 *	1.555 *
GB-M	10,317	0.506 *	0.944 *

* the linear regressions used to determine these parameters had correlation coefficients greater than 0.95.

**Table 4:** Fitting functions describing the average penetration depth of the indenter into bamboo as a function of the applied load (L), along with their primitives.

Specimens	Cubic Fit	Adherence Function
GB-0 *	102.07 + 391.16L − 846.03$L^{2}$ + 815.91$L^{3}$	102.07L + 195.58$L^{2}$ − 282.01$L^{3}$ + 203.98$L^{4}$ + C
GB-W *	104.28 + 449.57L− 879.03$L^{2}$ + 807.82$L^{3}$	104.28L + 224.79$L^{2}$ − 293.01$L^{3}$ + 201.96$L^{4}$ + C
GB-M *	101.98 + 487.65L − 1128.12$L^{2}$ + 1139.11$L^{3}$	101.98L + 243.83$L^{2}$ − 376.04$L^{3}$ + 284.78$L^{4}$ + C

* the linear regressions used to determine these parameters had correlation coefficients greater than 0.99.

## Data Availability

The raw data supporting the conclusions of this article will be made available by the authors upon request.
